# The ACPGBI AI taskforce report: A mixed‐methods roadmap for AI in colorectal surgery

**DOI:** 10.1111/codi.70232

**Published:** 2025-09-16

**Authors:** James M. Kinross, Kyle Lam, Andrew Yiu, Katie Adams, Kiran Altaf, Elaine Burns, Mindy Duffourc, Nicola Eardley, Charles Evans, Stamatia Giannarou, Laura Hancock, Victoria Hu, Ahsan Javed, Shivank Khare, Evangelos Mazomenos, Linnet McGeever, Susan Moug, Piero Nastro, Sebastien Ourselin, Subramanian Ramamoorthy, Campbell Roxburgh, Catherine Simister, Danail Stoyanov, Gregory Thomas, Pietro Valdastri, Marcus Vass, Dale Vimalachandran, Tom Vercauteren, Justin Davies

**Affiliations:** ^1^ Department of Surgery and Cancer Imperial College London London UK; ^2^ Department of Colorectal Surgery Guy's and St Thomas' NHS Foundation Trust London UK; ^3^ Department of Colorectal Surgery Liverpool University Hospitals NHS Foundation Trust Liverpool UK; ^4^ Department of Colorectal Surgery St Mark's ‐ The National Bowel Hospital London UK; ^5^ Faculty of Law Maastricht University Maastricht the Netherlands; ^6^ Faculty of Health, Medicine and Society University of Chester Chester UK; ^7^ Department of Colorectal Surgery University Hospitals Coventry and Warwickshire NHS Trust Coventry UK; ^8^ Department of Colorectal Surgery Manchester University NHS Foundation Trust Manchester UK; ^9^ Department of Medical Physics and Biomedical Engineering University College London London UK; ^10^ Department of Colorectal Surgery NHS Forth Valley Stirling Scotland; ^11^ Department of Colorectal Surgery Royal Alexandra Hospital Paisley Scotland; ^12^ Department of Colorectal Surgery Golden Jubilee University National Hospital Clydebank UK; ^13^ Department of Colorectal Surgery Dartford and Gravesham NHS Trust Dartford UK; ^14^ School of Biomedical Engineering & Imaging Sciences King's College London London UK; ^15^ School of Informatics University of Edinburgh Edinburgh UK; ^16^ School of Cancer Sciences University of Glasgow Glasgow UK; ^17^ School of Electronic and Electrical Engineering University of Leeds Leeds UK; ^18^ Osborne Clarke LLP London UK; ^19^ Department of Molecular and Clinical Cancer Medicine University of Liverpool Liverpool UK; ^20^ Department of Colorectal Surgery Cambridge University Hospitals NHS Foundation Trust Cambridge UK; ^21^ School of Clinical Medicine University of Cambridge Cambridge UK

**Keywords:** artificial intelligence, foundational datasets, generative AI, implementation strategy, machine learning, surgical education, surgical innovation

## Abstract

**Aim:**

The ACPGBI has commissioned a taskforce to devise a strategy for integrating artificial intelligence (AI) into colorectal surgery. This report aims to (i) map current AI adoption amongst UK colorectal surgeons; (ii) evaluate knowledge, attitudes, perceptions and experience of AI technologies; and (iii) establish priority recommendations to drive innovation across the specialty.

**Methods:**

A prospective 45‐item questionnaire was circulated to the ACPGBI membership. Questionnaire findings were explored at a multidisciplinary round table of surgeons, allied professionals, computer scientists and lawyers. Strategic recommendations were then generated.

**Results:**

122 members responded (75.4% consultants; 72.1% male; modal age 41–50 years). Although 43.5% used AI daily, only one third said they could explain key concepts within AI. 86.9% anticipated routine future‐AI use, with documentation and imaging ranked highest. 88.5% endorsed formal AI training. Major obstacles were unclear regulation, cost, medicolegal liability and professional or patient distrust. The round table generated 17 recommendations across clinical, educational and research domains and a ten‐point action plan, including the establishment of a Colorectal AI Committee and the creation of an open‐source colorectal foundational data initiative.

**Conclusion:**

This taskforce report combines questionnaire insights from the ACPGBI membership and expert debate into 17 key recommendations and a ten‐point action plan that will set the direction of future colorectal AI practice. The objective is to establish a framework through which colorectal surgical practice can be augmented by safe, trustworthy AI.

## INTRODUCTION

**FIGURE 1:**
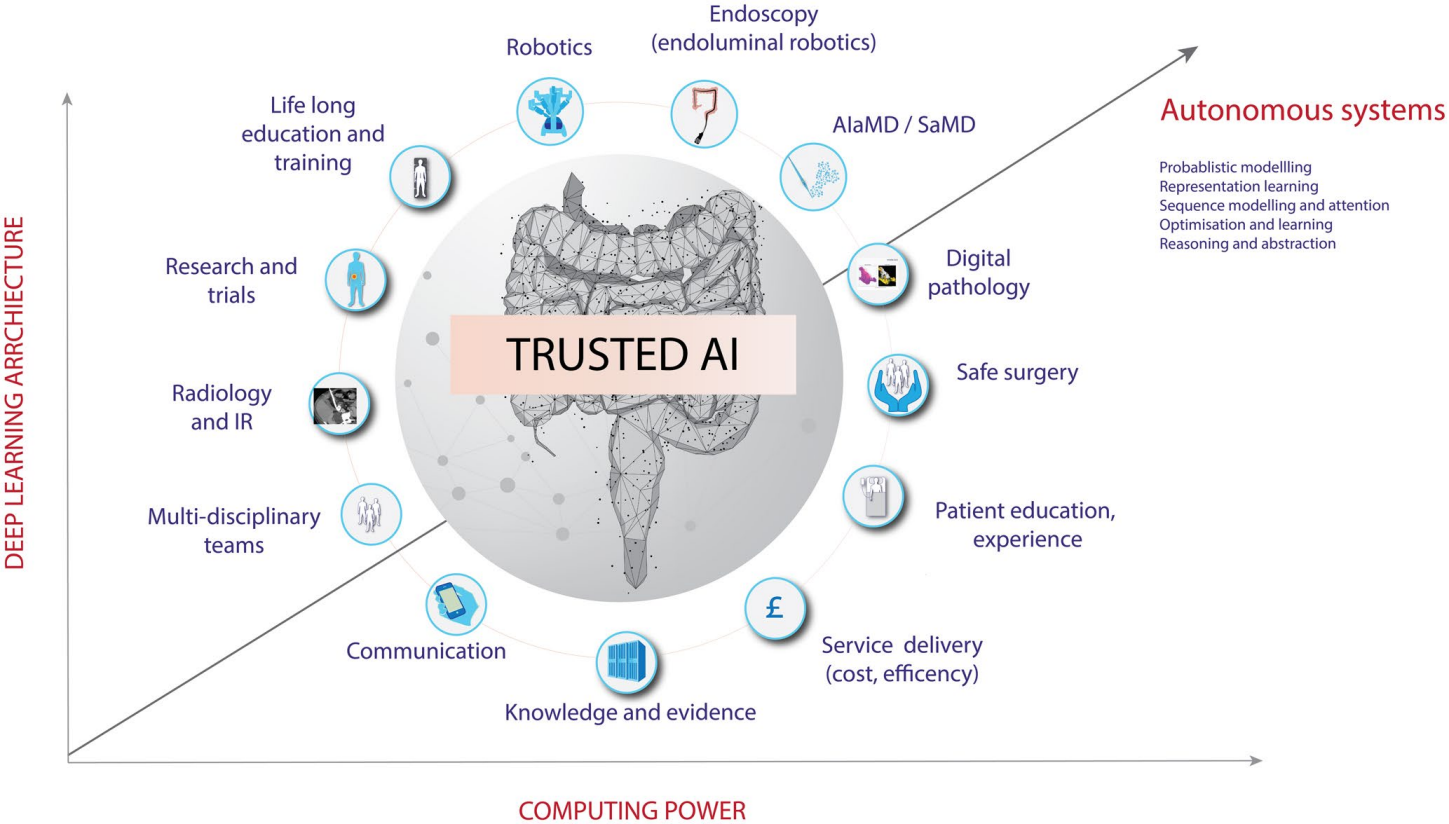
Challenges in AI adoption in colorectal practice. Practitioners have multidisciplinary careers that leverage foundational data sets from multiple specialities. The adoption of AI is being driven by rapid advances in generative AI learning architecture, computing power and advances in methodologies that are accelerating semi‐autonomous practice and a new future of surgical work that demands attention.

Artificial Intelligence (AI) refers to advanced machine‐based systems that replicate human learning, problem‐solving and decision‐making to achieve given goals or answer given questions [1]. This is not an incremental innovation, but a cross‐cutting generational technology that promises a transformation in general surgical practice and a radical departure from analogue colorectal surgery. Rapid advances in affordable computing power [2], deep learning architecture (e.g. transformers) [3], pre‐training technologies (e.g. visual representation learning) [4, 5], data access [6], and reasoning [7], mean that AI‐enhanced surgical practice is now theoretically achievable [8]. AI‐enhanced surgery does not just refer to surgical endoscopes or robots [9, 10, 11], but processes and systems that extend across the entire patient journey and across all scales of surgical practice (Figure 1).

The ambition of the UK government is for generative AI (GenAI) to revolutionise the operational efficiency of the NHS and to generate revenue through the commercial exploitation of NHS data [12]. Promising to invest £1 billion to increase the UK's compute capacity twentyfold in the next 5 years, the government has committed to investing in the adoption of AI to promote national economic growth [12, 13]. Professional medical associations, such as radiology and pathology, that possess access to foundational datasets, which allow the training of vast AI models, have already published guidance to promote AI adoption [14, 15]. The government's strategy, in the context of a global race for AI dominance, means that colorectal surgical services in the UK have a significant opportunity to embrace this technology, and with‐it potential to transform the future of colorectal work for the better.

Despite the extraordinary potential for AI systems to improve patient outcomes and surgical practice, surgical adoption of AI has, however, been slow relative to other specialities and industries. The Royal Colleges of Surgeons of England are moving to address this gap through the Commission on the Future of Surgery [16, 17]. International surgical colleges and associations have released AI policy statements [18, 19]. However, these have also been limited in scope. There is, therefore, an urgent need for a colorectal subspeciality strategy for safe AI adoption that meets the specific requirements of its patients and its workforce (Figure 1).

This is also time critical; the AI revolution is happening at incredible speed, bringing with‐it significant challenges [20, 21]. The potential for malicious use of AI and cybersecurity breaches poses immediate threats [22]. Further challenges exist in bias and underrepresentation of patient and surgeon cohorts within the AI models that are integrated into tools for colorectal surgery and colorectal diseases [23]. Some challenges are unprecedented; for the first time in history, the work of colorectal surgeons and other healthcare practitioners will be constantly measured to generate data points in AI development and use, which threatens to establish an unhealthy culture of surveillance in the workplace. Additionally, an increasingly automated colorectal workforce may result in practitioners losing their jobs. Beyond changes to the organisation of surgical workflows and culture, existential risks stemming from algorithmic failure or diminished therapeutic autonomy and control in high‐risk surgical environments also present new ethical obstacles that require immediate attention. But perhaps the greatest risk comes from failing to adequately manage the transition to AI‐enabled colorectal practice, as rudderless AI implementation will inevitably widen existing health inequalities.

The Association of Coloproctology of Great Britain and Ireland (ACPGBI) commissioned a taskforce to develop a strategy to integrate AI into colorectal surgery in a safe, ethical and responsible manner [24]. This report aims to provide an overview of the current adoption of AI amongst colorectal surgeons, assess current knowledge, attitudes, perceptions and experiences with these technologies, and establish priorities to prepare for a future where AI technologies (Table 1), from large language models to surgical robots, enhance patient outcomes, support clinicians and drive innovation in colorectal surgery. This is based on a mixed methods, qualitative analysis using a prospective questionnaire of the ACPGBI membership. A multidisciplinary team consisting of surgeons, allied healthcare professionals, domain experts and lawyers then convened for a structured discussion on the future strategy for AI adoption. This concluded with a series of recommendations that aim to establish UK colorectal surgery as a leader within the field of surgical AI adoption and innovation.

**TABLE 1:** Definition of key AI terms.

Term	Definition
Artificial Intelligence (AI)	Advanced machine‐based systems that replicate human learning, problem‐solving and decision‐making to achieve given goals or answer given questions.
Machine Learning (ML)	Subdomain of AI, algorithms and statistical models are used to analyse and infer patterns in data without explicit instruction.
Deep Learning (DL)	Subset of ML, multilevel artificial neural networks processing extract progressively higher‐level features from data.
Computer Vision (CV)	Computers analyse and interpret digital images and videos, replicating a human seeing.
Generative AI (GAI)	Subset of DL, can generate original content such as text, images, videos or code and presents this in a way understandable to humans.
Foundation model	Subset of GAI, can be applied to a wide range of use cases with minimal additional training.
Foundational datasets	Vast datasets upon which foundation models can be trained.
Large language model (LLM)	Subset of GAI, generates original text, trained from very large datasets.
AIaMD	Artificial Intelligence as a Medical Device.
SaMD	Software as a Medical Device.

## METHOD

### Questionnaire

A web‐based questionnaire was designed with reference to the NASSS (non‐adoption, abandonment, scale up, spread, sustainability) framework, which has been widely used to examine the uptake of emerging technologies [25]. The questionnaire aimed to capture the current landscape of AI amongst the ACPGBI membership, assessing respondents' current knowledge, awareness and utilisation of AI within colorectal surgical practice, and associated attitudes, perceived barriers and concerns (Supplementary Material 1). It also examined respondents' views on AI use within research and industry. It was distributed to all members of the ACPGBI, including colorectal consultants, associate specialists, trainees, nurses, technicians and healthcare undergraduates. Responses were collected via Qualtrics (Seattle, WA) from 17th February to 14th April 2025. Descriptive statistics were performed, with Chi squared and Fisher's exact tests used to determine group differences. Qualitative free text responses underwent thematic analysis by VH, AY and KL with an iterative coding process to identify themes.

### Colorectal/AI expert round table discussion

A hybrid round table discussion was hosted by the ACPGBI at the Royal College of Surgeons of England and online via Microsoft Teams on 28th April 2025. Preceding the meeting, participants were provided with current governmental policy, key literature and existing international surgical colleges and associations policies on AI implementation. Participants completed an initial scoping questionnaire prior to the discussion to allow ideas to be generated in advance and to ensure attendees had considered key challenges associated with AI implementation ahead of the meeting. Members of the ACPGBI Executive Council, ACPGBI AI Taskforce and academic and legal AI experts then convened to consider the results of the questionnaire and to define strategic priorities for the use of AI in colorectal surgery across clinical, education and research domains. A transcript of the discussion was analysed and an inductive thematic analysis conducted (AY and KL) to generate key aims across each domain to be actioned by the ACPGBI.

Ethical approval and informed consent were not required for this study as questionnaire respondents and interviewees are not considered research participants by the NHS Health Research Authority.

## RESULTS

### Questionnaire

**FIGURE 2:**
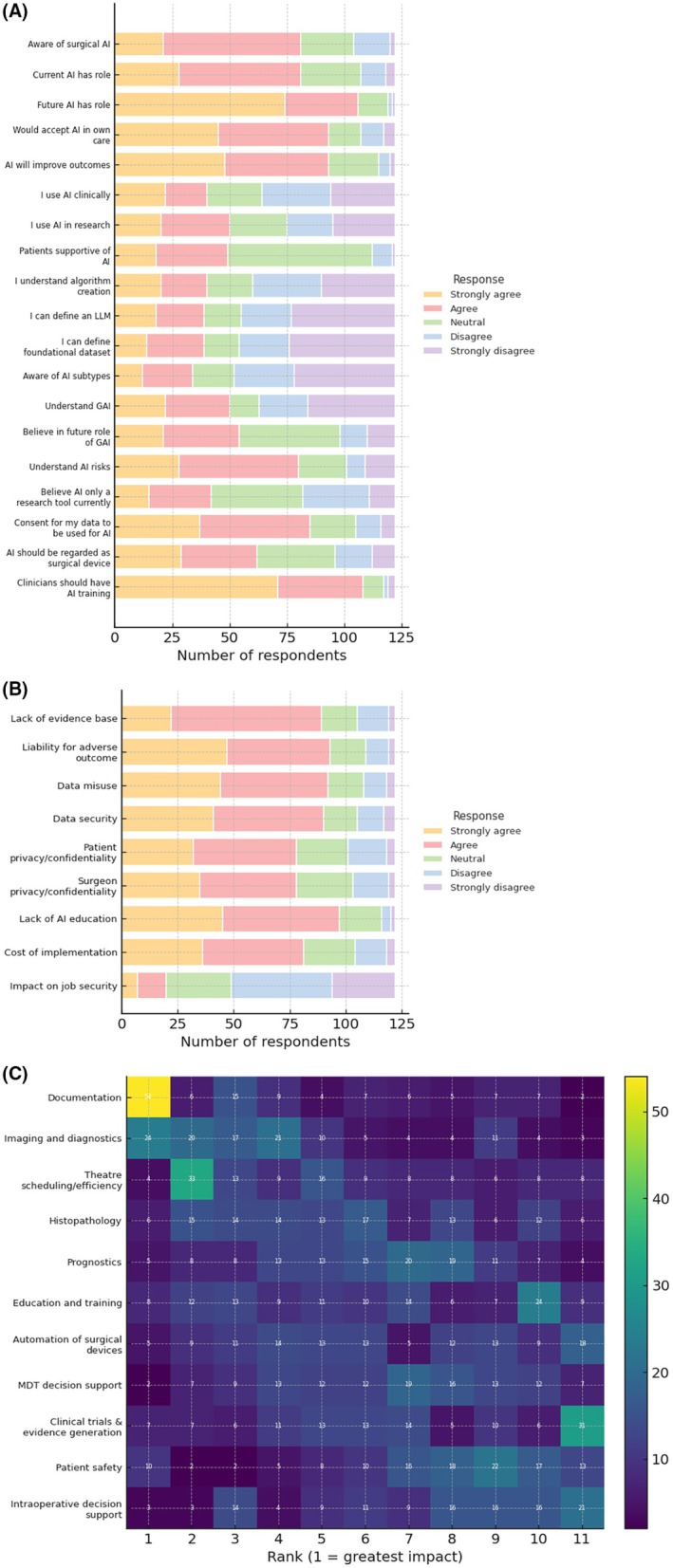
Findings from the ACPGBI AI questionnaire. A: Awareness and Knowledge of AI in Surgery; B: Concerns around AI in surgery; C: Domains where AI could positively impact patient care.

122/200 (61.0%) complete questionnaires were collected over the 8‐week period, with an additional 78 questionnaires submitted but incomplete and therefore not included in the analysis. Of the respondents, participants were more commonly male (72.1%), aged between 41 and 50 (36.9%), white (57.4%) and of consultant grade (75.4%). All respondents were over 30 years old and were of consultant, registrar or speciality or associate specialist grade. Data are summarised in Figure 2.

#### Digital literacy, use and knowledge of AI

82% of respondents somewhat or strongly agreed with feeling confident using technology in their day‐to‐day lives, with just under half using AI daily. 43.5% of respondents agreed they were knowledgeable about AI; however, fewer agreed they were able to explain AI concepts (32% for LLMs and foundational datasets, 41% for GenAI) and understand algorithm creation (32.8%) and AI methodologies (27.84%). 66.4% of respondents reported awareness of AI applications. 32.8% of respondents reported AI use in clinical settings and 41% of respondents reported AI use for research.

Subgroup analysis demonstrated that male participants reported significantly greater willingness to utilise technology and higher awareness of AI applications within surgery than female participants (*p* = 0.0011). Younger respondents were also significantly more likely to report willingness to utilise technology (*p* < 0.0001). No statistically significant differences were observed across ethnic groups or professional grades.

#### Attitudes toward AI in surgery

Participants demonstrated positive attitudes regarding AI integration. 66.4% of respondents believed that current AI has a role within surgery, whilst 86.9% of respondents believed AI will play a role in the future. 76.2% of respondents agreed that AI would improve patient outcomes. AI‐assisted documentation and imaging and diagnostics were ranked as having the greatest positive impact on care, whilst AI‐assisted intraoperative decision support and patient safety applications were ranked as having a lesser impact.

#### Barriers, concerns and trust

81.1% respondents believed there will be resistance to AI in surgery, with key barriers including lack of regulatory guidance (86.1% agreed), financial cost (78.7% agreed) and distrust amongst surgeons (72.1% agreed) and patients (66.4% agreed). Participants had concerns around liability in the event of adverse outcomes (76.2% agreed). Trust in AI was varied across different applications, with support for administrative tasks scoring higher than clinical decision‐making adjuncts. 79.5% respondents agreed a lack of AI education was a key barrier to adoption, and 88.5% respondents agreed that clinicians should receive formal training on the safe use of AI.

#### AI in research, commercial development and industry

27.9% of respondents reported using AI in surgical research, and all technologies were early and reported as being Technology Readiness Level (TRL) [26] 3 or less. 41.2% of this research activity contributed to foundational data set creation and 52.9% to algorithm development, with only a minority devoted to ethics or policy research in AI. Opinions regarding the role of industry were mixed, with 38.5% of respondents agreeing that industry is a trustworthy partner and 23.7% of respondents agreeing that industry shares data appropriately.

### Colorectal/AI expert round table discussion

The round table discussion was attended by ten consultant colorectal surgeons, two colorectal registrars, one colorectal nurse, four computer scientists and two lawyers with a special interest in AI governance and implementation.

**FIGURE 3:**
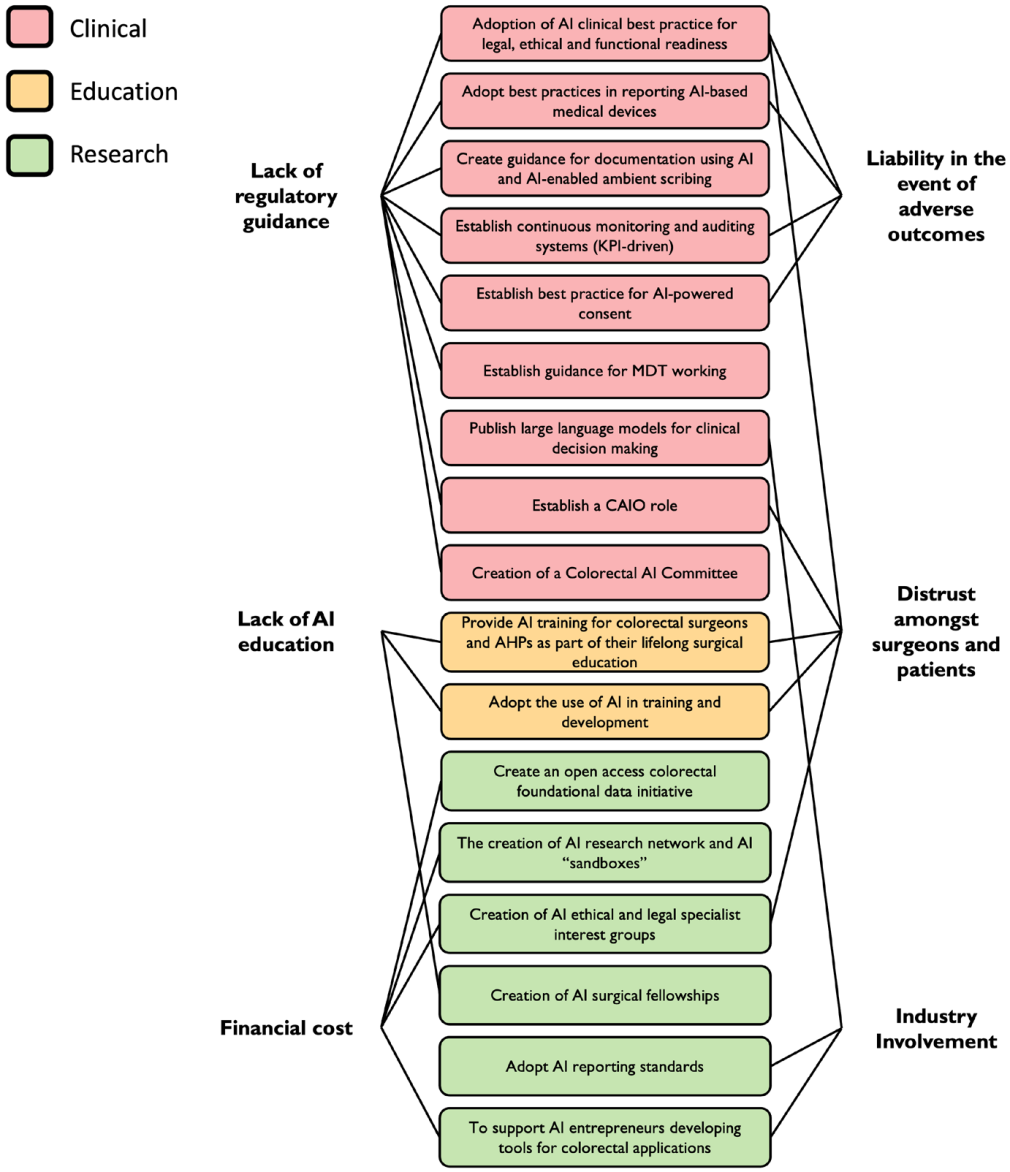
Barriers to the adoption of AI in colorectal surgery (black) and recommendations to address these across clinical (red), educational (yellow) and research (green) domains.

Given the breadth of AI innovation within colorectal surgery, the discussion was structured across clinical, educational and research domains. The panel was asked to help define barriers to the adoption of AI (Figure 3), how to maintain trust, and how to identify areas of impact and importance for colorectal surgeons. The following recommendations were strongly supported by the questionnaire data and the round table discussion.

#### Clinical recommendations

##### Recommendation 1: Adoption of AI clinical best practices for legal, ethical and functional readiness

Colorectal as a specialty should acknowledge and adopt UK best practices for AI use in clinical practice. This means following emerging UK [27] (and EU [28]) legislation and regulatory guidance on AI and established best practices that promote:

- Safety, Security and Robustness
- Appropriate Transparency and Explainability
- Fairness (bias, discrimination and inclusion)
- Accountability and Governance
- Contestability and redress (the ability to challenge algorithms)
- Sustainability – strategies for optimising data and resource utilisation, storage and maintenance and minimising the carbon footprints of AI systems.

This approach should form the main pillars of all future work in this domain. However, because AI regulation is rapidly evolving at national and international levels [29, 30, 31], the Colorectal community must be informed about the most important changes that relate to colorectal practice. It must also be proactive in shaping national and international strategies for AI policy in surgery. For example, advocating for the adoption of standards for AI as a medical device (AIaMD) and Software as a Medical Device (SaMD) that are uniquely tailored to colorectal surgery AI applications [32]. The Medicines and Healthcare Products Regulatory Agency (MHRA) is in the process of reforming the existing regulatory framework for SaMD to improve regulatory clarity and patient safety and responsible AI innovation [29]. MHRA's approach to collaboration both internationally (i.e. via the International Medical Device Regulators Forum (IMDRF)) and across UK government and industry (i.e. via the Multi Agency Advisory Service) will help ensure that the processes for validation, certification and ongoing monitoring will help to standardise AI adoption and ensure regulatory clarity [29]. This presents an opportunity to contribute to ongoing legislative and policy efforts to modernise frameworks governing AI and respond with timely information and best practice guidelines for its members.

##### Recommendation 2: Creation of a colorectal AI committee

Colorectal surgery should establish a dedicated AI committee to provide centralised leadership on AI‐related issues for its members in response to the questionnaire outcomes. Whilst there will initially be some overlap with established committees (such as robotics), AI is a cross‐cutting and rapidly evolving theme that urgently requires subject matter expertise to help shape strategy and policy. The committee will have a remit to coordinate all AI‐related activities, offering strategic guidance on AI adoption, facilitating AI education across the Colorectal community, the development of best practices and ensuring adopted AI is compliant with regulatory, legal and ethical standards and with General Medical Council (GMC) good clinical practice guidelines.

As AI becomes more prevalent in surgery, it is likely this AI committee will need to work with lawmakers creating legislation to protect patients, surgeons and allied healthcare professionals. Beyond medical device regulation, legal risk management and liability laws are also part of the larger AI governance ecosystem and a position in AI adoption, including cybersecurity, should be established. The future of colorectal surgical work will also change dramatically as we inevitably move toward automated practice. Whilst automation has the potential to reduce administrative burden and improve efficiency, the committee must advocate for the surgical workforce to ensure its best interests are maintained through this transition.

Finally, the committee should make a statement about how colorectal data assets (policies, guidelines and the Colorectal Disease journal) should be leveraged and used by AI models (e.g. LLMs) and acknowledge the role of openly sharing datasets in accelerating the development of AI tools. If delivered correctly, these AI‐based tools can be used for the creation, distribution and communication of this policy.

##### Recommendation 3: Establish a chief AI officer (CAIO) role

The Colorectal community should adopt a CAIO with suitable domain expertise and qualifications (akin to a Chief Information Officer (CIO)). Their role will be to provide strategic leadership on AI adoption. The role will also provide oversight for ethical and governance issues relating to AI in colorectal surgery, the assessment and creation of evidence‐based best practices for AI adoption, and the assessment of novel AI‐enabled technologies for use in colorectal surgery. The role should also serve as the primary liaison for the Colorectal community with wider stakeholders including governmental bodies, academic institutions and industry partners and the Royal Colleges of Surgeons. This post will be critical for ensuring national AI initiatives are integrated with our sister organisations in Europe (ESCP), the USA (ASCRS) and Australia and New Zealand (CSSANZ). Similarly, a CAIO would have responsibility for developing regulations and guidance on AI in surgery more broadly, with national and international policy makers, to ensure colorectal services, patients and providers are protected and prioritised. Delivery of AI initiatives at a local level will require coordination between the CAIO and Clinical CIOs, Caldicott Guardians and existing digital leads to avoid governance siloes.

##### Recommendation 4: Establish continuous monitoring and auditing systems (KPI‐driven)

Rigorous and continuous monitoring systems based on Key Performance Indicators (KPIs) to evaluate performance, effectiveness and safety of post‐market AI‐based medical devices should be established. Regular auditing will ensure adherence to predefined standards, detect deviations early and enable rapid corrective actions, therefore maintaining high‐quality clinical outcomes and building clinician and patient trust in AI technologies. This would require ongoing and iterative communication between developers, deployers and medical experts. It is feasible that much of this could be automated through AI agents and automated processes, developed with input from domain expertise, and this work could be overseen by the AI committee. In time, the development of a proprietary comprehensive colorectal AI Use ‘Checklist’ should be considered to assess rigorously the readiness of any AI‐based medical devices being developed for clinical integration into colorectal services and technologies.

##### Recommendation 5: Adopt best practices in reporting for AI‐based medical devices

Colorectal surgeons should be made aware of the developmental stage of AI‐based medical devices (experimental, developmental, validated) being used in clinical practice. When these tools impact patient self‐determination and autonomy in their medical treatment, for example by changing treatment risk profile, patients should also receive the information needed to maintain autonomy. Industry, small and medium‐sized enterprises (SMEs) and academics should be supported to make clear and explicit labels for the state of maturity of AI technologies and their readiness for clinical use. This status must also be transparently disclosed to patients so they are able to provide informed consent and to facilitate appropriate clinical expectations. Colorectal surgeons should follow international guidance when trialling AI instruments, to ensure that novel AI‐enabled devices or algorithms are standardised and scalable across the intended patient population. A good example of this is the FUTURE‐AI international consensus guidelines for trustworthy and deployable artificial intelligence in healthcare [33]. This will increase clinician and patient trust, reduce patient risk and allow for streamlined clinical adoption processes. Meeting these goals requires the development of a novel governance framework within the association in the form of best practices.

##### Recommendation 6: Create guidance for documentation using AI and AI‐enabled ambient scribing

The questionnaire demonstrated strong evidence that respondents felt generative AI was going to have the greatest impact in documentation (Figure 2C). The Colorectal community is recommended to follow best practice guidance issued by the NHS [34]. Clear, comprehensive documentation of AI contributions in clinical records should be mandated to maintain transparency and medicolegal clarity as per GMC guidance. Detailed documentation must explicitly indicate the role and extent of AI involvement in clinical decision‐making processes and treatments. Users of GenAI tools (such as ChatGPT) for clinical record keeping must retain responsibility for the content they input into the medical record. No patient confidential or identifiable data should be placed into a non‐regulated large language model, and surgeons should comply with UK General Data Protection Regulation (UK GDPR), follow standard recommendations on good cyber security practice issued by the National Security Cyber Centre (NSCC) [35] and utilise the NHS's Data Security and Protection Toolkit [36]. The Colorectal community should also be aware of when AI‐driven ambient scribes qualify as medical devices, for example those used for medical summarisation, and thus require a UK Conformity Assessed (UKCA) certificate and potential training [34].

##### Recommendation 7: Publish evidence for large language models (LLMs) for clinical decision‐making

These tools are extremely powerful and they are rapidly being developed for surgical decision support [37], and multiple other use cases in colorectal surgery such as pathology interpretation, patient advice and the interpretation of electronic healthcare records [38, 39, 40]. However, publicly available LLM‐driven GenAI systems should be used with caution, and they are currently unsafe for use in direct clinical care. This is because they are not regulated medical devices and they lack explainability and veracity. They are prone to hallucination and vulnerable to ‘data poisoning’ attacks [41]. This means they may provide misleading or incorrect information to clinicians and patients that can be harmful. Like any other medical device, when GenAI tools are used for clinical purposes, they require robust validation and regulatory approval before being used for this purpose in direct colorectal care. Current approaches for evaluating the use of LLMs and other AI methodologies used in research and publications are listed in Table 2.

**TABLE 2:** Summary of AI research consensus statements and guidelines.

Name	Year	Scope	Checklist / key items
SPIRIT‐AI [42]	2020	Clinical trial protocols involving AI	Extension of SPIRIT (Standard Protocol Items: Recommendations for Interventional Trials); adds AI‐specific protocol elements (e.g. dataset curation, model versioning)
CONSORT‐AI [43]	2020	Reporting randomised trials involving AI	Extension of CONSORT; focuses on interventions using AI, covering 14 new AI‐related items (e.g. input data handling, human–AI interaction)
DECIDE‐AI [44]	2022	Early‐stage clinical evaluation of AI	Reporting guideline for Phase I/II‐like studies (before RCTs); covers implementation, learning curves, context
TRIPOD‐AI [45]	2023–24	Clinical prediction models using ML	Extension of TRIPOD for ML‐based models (regression and non‐regression); emphasises internal/external validation, calibration
TRIPOD‐LLM [46]	2024	Clinical prediction with LLMs	LLM‐specific extension; 19 main items +50 sub‐items (e.g. prompt design, hallucinations, temperature settings)
FUTURE‐AI [33]	2023	Trustworthy AI in healthcare	6 principles: Fairness, Universality, Traceability, Usability, Robustness, Explainability; 30 best practices
CLAIM‐AI [47]	2024	AI for diagnostic imaging	Extension of CLAIM for AI models; includes imaging datasets, preprocessing, performance metrics
REFORMS [48]	2023	General ML science across disciplines	32‐question checklist; focuses on transparency, validity, reproducibility
CANGARU [49]	2023	LLM use in research (cross‐discipline)	Draft Delphi‐guided guideline for using/disclosing LLMs (e.g. ChatGPT) in publications

##### Recommendation 8: Establish best practice for AI‐powered consent

AI could improve the consistency and comprehensiveness of information delivered during the consent process, address language and communication barriers and even offer empathy [50]. It is also now conceivable that AI‐powered consent is achievable; AI agents are technically able to have complete conversations with patients regarding their consent for surgical procedures. However, there is currently no legal precedent for AI consent, and surgeons' legal and ethical duties to provide informed consent cannot be delegated to AI. The Colorectal community should engage in work to understand the implications of AI in the consent process. Patients must be adequately consented if GenAI tools are to be used in their colorectal care in ways that impact their self‐determination and autonomy. Beyond the ethical and legal obligations governing consent for medical treatment and research, the use of AI in patient care also implicates information and consent obligations in connection with data protection law. A multilayered consent framework recommendation for distinguishing treatment, data use and sharing, and AI tool development is recommended. This would document patient consent across treatment, data usage and involvement in AI research or tool development.

Patient Liaison Groups should also actively collaborate in developing accessible, patient‐centric educational materials and consent templates regarding AI technologies used in colorectal surgery. There is a need for AI agents that can improve how we communicate complex issues in colorectal surgical practice to patients [40]. These tools could effectively address patient needs and concerns surrounding AI and data usage, promoting better understanding, informed patient participation and improved levels of acceptance for AI use in colorectal surgery and for education around colorectal disease in general [51]. The development of these tools through collaboration with charities, patient organisations and academic institutions working within this field is recommended.

##### Recommendation 9: Establish guidance for multidisciplinary team (MDT) working

AI offers a major opportunity to relieve the burden on UK colorectal MDTs and to improve and standardise the quality of decision‐making nationally for colorectal disease. Whilst evidence for its use in colorectal surgery remains limited [52], there is evidence that LLMs demonstrate concordance in the quality of information they provide to colorectal specialists working in multidisciplinary settings [53, 54]. AI could also be used for multimodal data collation and for providing decision support or prompts through AI agents. The AI committee should seek to establish best practice guidance on the use of AI tools in the MDT as an urgent priority and establish research outcomes to demonstrate their safety and efficacy.

#### Educational recommendations

##### Recommendation 10: Provide AI training for colorectal surgeons and allied healthcare professionals as part of their lifelong surgical education

The Royal College of Surgeons of England has issued a broad‐ranging report on the use of technology in enhanced surgical education [17]. Whilst this is comprehensive, this must now move further to ensure colorectal surgeons are trained in essential AI competencies, including technical background, ethical and regulatory considerations and clinical applications. The goal is the safe adoption of AI into clinical practice. This will be delivered through short courses or online resources. This training should form part of a CPD‐accredited AI certification programme in colorectal surgery. Certification will validate proficiency in colorectal AI applications and support ongoing professional development and career progression.

To ensure comprehensive, multidisciplinary adoption of AI, education programmes must also extend beyond surgeons to include nurses, allied health care professionals and administrators. Broadening AI literacy is essential to integrate AI effectively across colorectal surgery, subsequently enhancing teamwork and improving clinical workflows.

##### Recommendation 11. Adopt the use of AI in training and development

There is an urgent requirement for a set of ethical and governance frameworks for the appropriate use of trainee data for the development of AI algorithms [55]. Trainees must be made aware of how their data are being used, and training roles must be appropriately communicated in the creation of future‐AI algorithms that may be used to quantify or assess their progress.

LLMs have demonstrated an ability to pass Royal College of Surgeons membership [56] and postgraduate board examinations in surgery [57], although their performance is imperfect due to hallucination, fallible information synthesis and contextual blindness. A position is required on the use of these technologies by trainees and its members in the certification of the courses it runs for knowledge‐based assessments and in their use when creating academic content (e.g. scientific papers or conference abstracts) and patient‐centred outputs. This should culminate in the creation of an AI good practice statement for members when using generative AI tools for academic, research or educational purposes.

Educational studies have now been performed using AI technologies to assess core competencies in colorectal surgery (e.g. robotics and laparoscopic surgery); however, data suggest these approaches are not well validated and there is a lack of standardisation in their adoption [58]. Urologists have established a consensus statement for the ethical implications of AI on robotic training, concluding that data and privacy issues, transparency, biases, accountability and liabilities were risks [59]. This has yet to occur in colorectal surgery, and it is urgently needed. This is because the autonomous analysis of technical, team and cognitive performance, digital twins and world AI models are likely to radically transform the training landscape in the midterm. This is being expedited by the emergence of cloud‐based software for tele‐proctoring and video‐based autonomous analysis of technical competences. These are increasingly linked to robotic platforms (Intuitive, Medtronic and CMR all possess their own digital ecosystem) and because of this, it is feasible that if action is not taken promptly, training quality and access will be determined by a pseudo‐surgical‐feudalism.

The solution requires the development of a novel colorectal surgical AI pedagogy, validation instruments, quality assurance tools and processes that all manufacturers providing AI‐enabled surgical training platforms must meet for colorectal surgery. There is also the requirement for novel assessment tools for measuring surgical knowledge and skill set development in a semi‐autonomous environment. Whilst this may seem like a distant vision of future surgery, this nettle must be grasped now. If achieved, there is a very significant opportunity to radically reduce training times, improve training experiences and with‐it patient outcomes is significant.

#### Research recommendations

The questionnaire report suggests that whilst there are pockets of excellence in colorectal AI research across the country, this is happening in isolated units and at a small scale. The AI committee, working together with ACPGBI, should now establish research priorities for AI in the speciality and lobby the government for improved funding for colorectal surgical AI research. Research priorities have previously been mapped for digital surgery [60], and any future strategy must address three fundamental challenges: (1) The creation of open access, large scale, longitudinal foundational data sets for building colorectal surgical AI. (2) Establishing national AI research networks in colorectal surgery that can pilot and trial novel AIaMD technologies but can also address fundamental roadblocks to translational AI such as explainability, veracity or data hallucination. (3) Trust: Establish ethical and legal frameworks for safe AI research that addresses near term and future risks of autonomous systems (e.g. loss of control or malicious use).

##### Recommendation 12: Creation of an open‐access colorectal foundational data initiative

A major goal of the AI committee must be to support the creation of foundational data sets in colorectal surgery that will enable and support the continuous development of generative AI tools for colorectal surgical practice. Surgeons and data scientists should be actively supported to share videos and open‐access foundational data sets. If this does not happen, research datasets risk being locked in commercial siloes [61]. Creation of open research datasets and best practices in the sharing of foundational data sets, the most valuable of which is likely to initially be video, must be promoted [62, 63].

**FIGURE 4:**
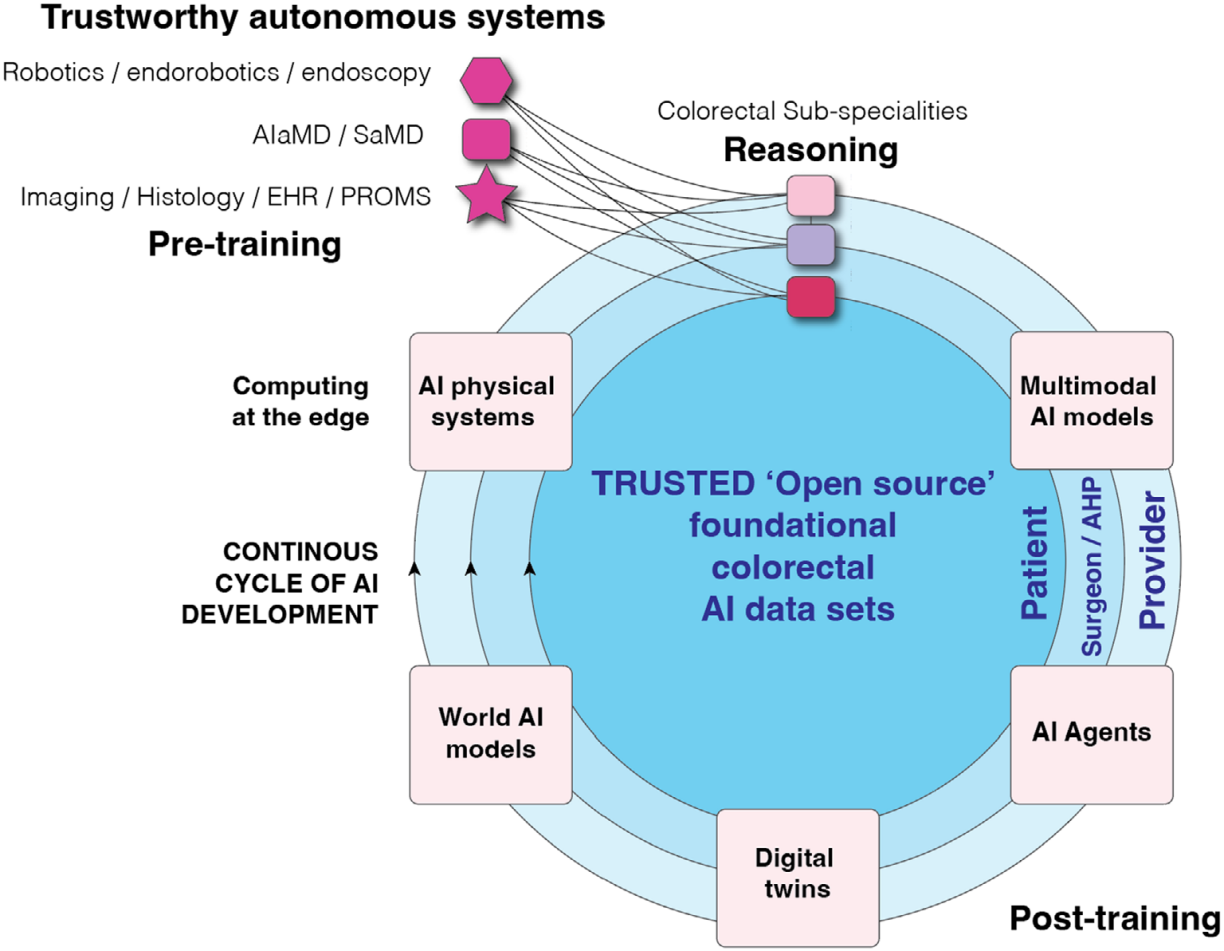
The data cycle necessary for driving AI adoption in colorectal surgery.

The round table panel noted that building foundational data sets is prohibitively expensive and likely beyond the remit of the ACPGBI. However, scalable foundational data sets are central to the safe and democratic development of AI systems (Figure 1). An open Colorectal data initiative will promote and advocate for the collection and sharing of high‐quality data. It was therefore recommended that data standards and ontologies upon which robust, longitudinal foundational AI data sets can be built in the future should be established (Figure 4). This also requires scalable and standardised governance structures, ethical frameworks, data standards and ontologies that ensure colorectal AI foundational models are open, high quality and large enough in scale to be useful. Proof of concept for this strategy has already been achieved by SAGES for video annotation [64], usage, structure and exploration [65].

Whilst all industry partners engaging in AI research in the UK must legally follow GDPR guidance on data protection, not all data is made available to researchers. More importantly, these data are being used to create algorithms that are financially valuable. The Colorectal community should therefore seek to work with companies that aggregate anonymised video, kinematic, experiential or behavioural foundational datasets, and negotiate data access for research purposes. A Colorectal AI initiative, that has clear objectives and deliverables, would be the first step in this process and this approach could serve as a cornerstone for AI tool development, validation and evaluation where all stakeholders can derive value. A key reference for this could be the new European Health Data Space (EHDS) [66] whereby the European Union aims to establish a common framework for the use and exchange of electronic health data.

##### Recommendation 13: The creation of an AI research network and AI ‘sandboxes’

The Colorectal community must aim to prevent repetition and siloed working. It must also help researchers overcome regulatory barriers for the rapid translation of AI innovation into clinical practice.

AI environments already exist in academic organisations, and if connected, they will provide safe and controlled spaces to rigorously test new AI tools under simulated or real‐world conditions for colorectal AI technologies. The colorectal community should therefore look build to build networks of AI laboratories that are actively involved in colorectal AI research to facilitate controlled, real‐world evaluation of AI technologies. They could encourage this by providing access to patients, clinicians and translational research expertise. This will address a major barrier in colorectal AI research by improving access to computing resources, hardware and expertise. This will also add significant value for industrial partners and SMEs who are performing research in this space. This network will require adequate resources as it will need to be actively managed, with available digital resources that promote an active community of AI researchers and their projects.

As part of this initiative, the Colorectal community should develop strategic collaborations with speciality associations to maximise patient benefit from AI and to promote access to domain expertise.

##### Recommendation 14: Creation of an AI ethical and legal specialist interest group

The Colorectal community should promote rigorous external validation protocols emphasising demographic equity checks to minimise biases and ensure representative datasets to promote ethical and legally responsible AI integration into colorectal practice. It must also establish best practices for explainability and veracity checking in surgical AI data sets and tools and promote research that is sustainable and safe. This should include the development of transparent shared‐benefit agreements with industry partners, ensuring fair and transparent data handling, equitable intellectual property rights and mutual benefit. These agreements will set out expectations, roles and standards and will act as a starting point for institutes seeking partnerships with industry. Finally, given that the future of AI in colorectal surgery is unclear, the roundtable panel advocated for the inclusion of ethical and legal research in any future‐AI strategy.

##### Recommendation 15: Creation of AI surgical fellowships

Just as the ACPGBI promotes excellence in postgraduate advanced training, surgeon‐scientist leadership should be promoted through AI surgical Fellowships. Dedicated AI Fellowships should be established to support sustained research initiatives as deemed a priority by the membership of the ACPGBI. These should encompass all aspects of the continuous AI development cycle (Figure 3) and they should encourage the trial and development of AI tools in training and direct clinical care. AI surgical fellows are envisaged to become future surgeon‐scientist leaders capable of advancing AI‐driven research and pushing forward strategic AI objectives.

##### Recommendation 16: Adopt AI reporting standards

Multiple consensus statements and guidelines have now been generated for the use of AI in research (Table 1). The colorectal community should adhere to best practice principles when designing and reporting AI research. Given that reporting standards are now being reported frequently, specific guidelines for specific study types should also be recommended.

##### Recommendation 17: To support AI entrepreneurs developing tools for colorectal applications

The UK possesses exceptional talent in computing and AI. The questionnaire suggests that many colorectal surgeons are involved in entrepreneurial activity as they seek to develop these tools for clinical impact. A ‘dragons' den’ format could be used to promote this activity and to connect entrepreneurs with funders and industry. It should also promote this activity within its research network and value it as a form of societal impact.

## DISCUSSION AND CONCLUSIONS

The ACPGBI AI Taskforce questionnaire and Colorectal/AI expert round table discussion is the first work to assess the current status of, and the strategic plan for, the adoption of AI in Colorectal surgery in Great Britain and Ireland.

It was noted from the questionnaire that 75% of respondents either strongly agreed or agreed that they understood the risks of AI. However, only 32% of respondents reported they could explain how large language models work, differing types of AI, or even how algorithms are developed. It is also notable that nearly 84% of respondents believed that their jobs were not at risk from AI (Figure 2B). It therefore appears that there is a disconnect between how surgeons think AI is going to impact clinical practice and their actual understanding of the technology.

Current AI guidelines from interventional specialities, with a focus on gastroenterology, were reviewed and discussed. The goal of the majority of AI‐based endoscopic technologies has focused on improving adenoma detection rates and increasing the area of visualisation of the colonic mucosa. This has culminated in the deployment of AI‐enabled Computer Aided Detection (CADe) platforms in endoscopy [67, 68], and a rapid growth in AI methodologies for Computer Aided Quality assurance (CAQ) that will impact on endoscopic training and service delivery [69]. In response, the UEG [70], EGE [71], ASGE [72], the AGA [73] have established guidance for AI in endoscopy, and the BSG has also established an AI taskforce to improve the speed of translation of AI technologies into clinical practice with a focus on inflammatory bowel disease [74]. NICE is now preparing guidance for CADe in endoscopy [16] as it becomes increasingly commonly used in clinical practice. Surgical endoscopists are making significant contributions to the evolution of AI guidelines in his field, and the Colorectal community must now establish its position for how it will leverage these technologies in surgical endoscopic training and endoluminal surgery.

Colorectal surgeons are also likely to use radiological [75, 76, 77] or histological [78] AI‐based tools in their clinical practice either for diagnostics or prognostics. A major challenge is that these innovations are being led by radiologists and histopathologists because they have prioritised access to foundational data sets, and they have developed a coherent strategy for the adoption of AI; both of their Royal Colleges have also published guidance for AI implementation.

Finally, the next generation of AI‐enabled tools being developed for intraoperative practice was summarised. These encompass the objective analysis of intraoperative imaging such as flurophores [79], real time intraoperative pathological diagnosis and cancer margin detection [80, 81], anatomy identification [82, 83, 84], operative harm prevention [85, 86] and clinical outcome prediction [87]. To date, these have largely remained proof of concept and many are occurring in small scale pilot studies as part of a ‘narrow AI’ strategy. The opportunity for the Colorectal community is to scale these technologies into a ‘broad AI’ approach, by creating a national strategy for intraoperative AI, based on a coordinated approach that promotes research collaboration and high‐quality trials powered to appropriate clinical outcomes.

The ACPGBI questionnaire has limitations in terms of its power and its demographic bias, but it has demonstrated that attitudes toward AI's future in surgery across the membership are positive, particularly for autonomous documentation and diagnostics. However, it also identified that key barriers around regulatory uncertainty, cost and liability concerns and revealed a widespread lack of AI education and trust, especially in clinical decision‐making contexts. Whilst the round table discussion focused more on the practical challenges of introducing AI into real‐world colorectal surgical practice, it also concluded that the Colorectal community must now urgently move to adopt AI into its core strategy for improving the quality of colorectal surgery in the UK and Ireland. This framework should be viewed as a starting point for this process, and it makes short‐, medium‐ and long‐term recommendations summarised below (Table 3) to that effect. The key message is that the role of the ACPGBI is to build trust for all stakeholders in surgical AI. If it can achieve this goal, colorectal surgery could lead the world in surgical AI adoption, with the potential to accelerate the improvement of outcomes for patients.

**TABLE 3:** Strategic 10‐Point Plan for Near‐, Mid‐ & Long‐ Term AI Adoption.

Short‐term AI adoption	1	Establish a colorectal AI committee
	2	Create a CAIO post
	3	Develop Training & Certification for AI usage
	4	Establish AI fellowships
Medium‐term AI Adoption	5	Create Ethical and Legal working group for AI
	6	Develop guidance for use of AI in clinical practice that align with national Standards and best practice (e.g. FUTURE‐AI).
Long‐term AI Adoption	7	Establish open‐source colorectal foundational data sets
	8	Develop national network of colorectal AI sandpits
	9	Develop colorectal entrepreneurship in AI
	10	Development of bespoke tools for monitoring, auditing and iterating using AI tools

## AUTHOR CONTRIBUTIONS

**James M. Kinross:** Conceptualization; methodology; supervision; project administration; writing – original draft; writing – review and editing; data curation; investigation. **Kyle Lam:** Writing – original draft; writing – review and editing; formal analysis; investigation; data curation. **Andrew Yiu:** Data curation; investigation; formal analysis; writing – original draft; writing – review and editing. **Katie Adams:** Investigation; writing – review and editing. **Kiran Altaf:** Investigation; writing – review and editing. **Elaine Burns:** Investigation; writing – review and editing. **Mindy Duffourc:** Investigation; writing – review and editing. **Nicola Eardley:** Investigation; writing – review and editing. **Charles Evans:** Investigation; writing – review and editing. **Stamatia Giannarou:** Investigation; writing – review and editing. **Laura Hancock:** Investigation; writing – review and editing. **Victoria Hu:** Investigation; writing – review and editing. **Ahsan Javed:** Investigation; writing – review and editing. **Shivank Khare:** Investigation; writing – review and editing. **Evangelos Mazomenos:** Investigation; writing – review and editing. **Linnet McGeever:** Investigation; writing – review and editing. **Susan Moug:** Investigation; writing – review and editing. **Piero Nastro:** Investigation; writing – review and editing. **Sebastien Ourselin:** Investigation; writing – review and editing. **Subramanian Ramamoorthy:** Investigation; writing – review and editing. **Campbell Roxburgh:** Investigation; writing – review and editing. **Catherine Simister:** Data curation; investigation; formal analysis; writing – review and editing. **Danail Stoyanov:** Investigation; writing – review and editing. **Gregory Thomas:** Investigation; writing – review and editing. **Pietro Valdastri:** Investigation; writing – review and editing. **Marcus Vass:** Investigation; writing – review and editing. **Dale Vimalachandran:** Investigation; writing – review and editing. **Tom Vercauteren:** Investigation; writing – review and editing. **Justin Davies:** Conceptualization; writing – review and editing; supervision.

## FUNDING INFORMATION

The authors received no specific funding for this work.

## CONFLICT OF INTEREST STATEMENT

TV is co‐founder and shareholder of Hypervision Surgical Ltd. JD is a Specialist Committee Member for the NICE Health Tech Programme Diagnostics Assessment for Artificial Intelligence software to help detect and characterise colorectal polyps.

## ETHICS STATEMENT

Not applicable.

## Supporting information


Data S1.

## Data Availability

The data that support the findings of this study are available from the corresponding author upon reasonable request.
