# Supplementary material for: The ACPGBI AI taskforce report: A mixed‐methods roadmap for AI in colorectal surgery
Source: Colorectal Dis. 2025 Sep 16;27(9):e70232. doi: 10.1111/codi.70232 (PMC12441474; doi:10.1111/codi.70232)
Supplement: Supplementary file 1 — Data S1. [file CODI-27-0-s001.docx]

**Supplementary Materials**

Supplementary Material 1: ACPGBI AI Taskforce Questionnaire

|  | *Strongly Agree* | *Somewhat Agree* | *Neither Agree nor Disagree* | *Somewhat Disagree* | *Strongly Disagree* |
| --- | --- | --- | --- | --- | --- |
| **Please describe your level of expertise in AI and technology:** |  |  |  |  |  |
| I feel confident using technology in my day to day life |  |  |  |  |  |
| I consider myself an early adopter of new technology |  |  |  |  |  |
| I use AI in my day to day life |  |  |  |  |  |
| I consider myself knowledgably about how AI works |  |  |  |  |  |
|  |  |  |  |  |  |
| **Please describe your awareness and knowledge of AI in surgery** |  |  |  |  |  |
| I am aware of AI applications within surgery |  |  |  |  |  |
| Current AI has a role within clinical surgery |  |  |  |  |  |
| Future AI will have a role within clinical surgery |  |  |  |  |  |
| I would accept the use of AI in my own personal care |  |  |  |  |  |
| AI will improve patient outcomes in surgery |  |  |  |  |  |
| I use AI in my clinical surgical practice |  |  |  |  |  |
| I use AI within a research setting |  |  |  |  |  |
| I believe that patients are supportive of the use of AI in surgery |  |  |  |  |  |
| I understand how AI algorithms are created |  |  |  |  |  |
| I can confidently explain what a LLM is and its limitations |  |  |  |  |  |
| I can confidently explain what a foundational dataset is an its importance to AI algorithm development |  |  |  |  |  |
| I'm aware of the different types of AI methodologies |  |  |  |  |  |
| I understand what the term generative AI means |  |  |  |  |  |
| I believe generative AI has a future role in surgery |  |  |  |  |  |
| I understand the potential risks of AI use in surgery |  |  |  |  |  |
| I believe that AI and surgery is currently only a research tool |  |  |  |  |  |
| I would consent to my own surgical practise being used to train surgical AI algorithms |  |  |  |  |  |
| AI should be regarded as a surgical device |  |  |  |  |  |
| Clinicians should receive formal training on how to use AI safely |  |  |  |  |  |
|  |  |  |  |  |  |
| **Perceived enablers and barriers to the use of surgical AI** |  |  |  |  |  |
| I believe there will be resistance to the use of AI in surgery |  |  |  |  |  |
|  |  |  |  |  |  |
| **Barriers to the application of AI in surgery include** |  |  |  |  |  |
| Removal of autonomy of surgeons |  |  |  |  |  |
| Lack of regulatory or monitoring guidelines |  |  |  |  |  |
| Surgeon distrust in AI |  |  |  |  |  |
| Patient distrust in AI |  |  |  |  |  |
| Financial cost of developing, implementing and evaluating AI |  |  |  |  |  |
|  |  |  |  |  |  |
| **Trust in AI** |  |  |  |  |  |
| I trust AI outputs as an adjunct to my own clinical decision making |  |  |  |  |  |
| I trust AI to perform administrative tasks more efficiently |  |  |  |  |  |
| I trust AI outputs as a means of highlighting clinical information |  |  |  |  |  |
|  |  |  |  |  |  |
| **Please answer the following questions on concerns around the use of surgical AI** |  |  |  |  |  |
| I'm concerned about the use of AI in surgery |  |  |  |  |  |
|  |  |  |  |  |  |
| **My concerns around the use of AI and surgery include** |  |  |  |  |  |
| Lack of evidence base |  |  |  |  |  |
| Liability in the event of an adverse outcome |  |  |  |  |  |
| Data misuse |  |  |  |  |  |
| Data security |  |  |  |  |  |
| Patient privacy and confidentiality |  |  |  |  |  |
| Surgeon privacy and confidentiality |  |  |  |  |  |
| Lack of AI education among surgeons |  |  |  |  |  |
| Cost of AI implementation in surgery |  |  |  |  |  |
| The impact it will have on my job security |  |  |  |  |  |
|  |  |  |  |  |  |
| **Please answer the following questions on industry and AI** |  |  |  |  |  |
| Industry can be trusted to deploy AI safely |  |  |  |  |  |
| Industry shares its data effectively on its use of AI within surgeons |  |  |  |  |  |
| Industry should take the lead in the development of surgical AI algorithms |  |  |  |  |  |

|  | *Yes* | *No* |
| --- | --- | --- |
| **AI and surgical research** |  |  |
| I use AI in my surgical research |  |  |
| Please state the field of your surgical research | *Foundational data set creation* | |
|  | *Robotics* | |
|  | *Imaging* | |
|  | *Algorithm development and computer science* | |
|  | *Clinical trials* | |
|  | *AI ethics/policy/legal* | |
|  | *Other* | |
| Are you a co-founder of an AI start up? |  |  |
| Are you collaborating with an industrial partner? |  |  |

| **Please place the following domains in order where AI is likely to have the greatest positive impact on patient care** |
| --- |
| Documentation (operative or clinical notes) |
| Theatre scheduling/efficiency |
| Intraoperative decision support |
| Automation of surgical devices |
| MDT decision support |
| Prognostics |
| Patient safety |
| Histopathology |
| Imaging and diagnostics |
| Education and training |
| Clinical trials and evidence generation |
